# Development and Validation of Natural Language Processing (NLP)‐Based Risk Prediction Model for Cognitive Impairment in Geriatric Patients

**DOI:** 10.1002/alz.088942

**Published:** 2025-01-09

**Authors:** Laili Soleimani, FU‐YUAN CHENG, Mary Sano, Arash Kia

**Affiliations:** ^1^ Icahn School of Medicine at Mount Sinai, New York, NY USA; ^2^ James J Peters VAMC, Bronx, NY USA

## Abstract

**Background:**

Dementia poses a significant global crisis, yet 60% of cases go undetected, particularly among specific sub‐populations. Timely diagnosis is crucial for implementing early intervention strategies. Challenges of current screening tools (e.g., Montreal Cognitive Assessment (MOCA) and Mini‐mental Status Exam (MMSE)) include their rule‐based nature, time‐consuming administration and potential bias against individuals with lower education or different socioeconomic backgrounds.

**Method:**

In this study, we created a patient cohort within Mount Sinai Health System, identifying individuals at high‐risk for cognitive impairment using MMSE scores 23 and below. Extracting clinical notes from inpatient and outpatient settings, we applied specific sampling logic to select the last five notes for each patient before their MMSE. Utilizing word‐embedding techniques, we vectorized sentences and input these vectors into a deep neural network for Named Entity Recognition to extract clinical concepts (e.g diagnosis, signs and symptoms, medications). A psychiatrist reviewed and selected 337 features based on their frequency in the cohort and clinical relevance. Using these features, we created a map to generate feature vectors, and following a train‐test split, 70% of the data was used to train a classifier using the random forest algorithm. The remaining 30% of the cohort was employed for validation purposes.

**Result:**

Out of 4,366 patients, with documented MMSE scores from 2011 to 2023, 73% had one MMSE, and 29% scored below 24. The developed model demonstrated an AUC ROC of 0.93. At a threshold of 0.5, it showed a sensitivity of 0.86 and specificity of 0.85 in the validation set. On the test set, the AUC ROC was 0.68, with a sensitivity of 0.62 and specificity of 0.63 at a threshold of 0.5.

**Conclusion:**

The development and validation of this digital tool exemplify translational research that leverages Real‐World Data (RWD) from EMR. The tool aims to assist clinicians in efficient dementia detection across different stages of the disease. Our next phase involves optimizing the feature engineering, fine‐tune the model, and deploying it in the production environment. This will facilitate automated screening of geriatric patients during clinical encounters at Mount Sinai Hospital, allowing us to measure the tool’s prospective performance.

